# Patient Engagement and its Evaluation Tools– Current Challenges and Future Directions: Comment on "Metrics and Evaluation Tools for Patient Engagement in Healthcare Organization- and System-Level Decision-Making: A Systematic Review"

**DOI:** 10.15171/ijhpm.2019.16

**Published:** 2019-03-19

**Authors:** Samira Abbasgholizadeh Rahimi, Hervé Tchala Vignon Zomahoun, France Légaré

**Affiliations:** ^1^ Department of Family Medicine, Faculty of Medicine, McGill University, Montreal, QC, Canada.; ^2^ Health and Social Services Systems, Knowledge Translation and Implementation Component of the Quebec SPOR-SUPPORT Unit, Université Laval, Quebec, QC, Canada.; ^3^ Université Laval Primary Care Research Centre (CERSSPL-UL), Université Laval, Quebec, QC, Canada.; ^4^ Department of Family Medicine and Emergency Medicine, Faculty of Medicine, Université Laval, Quebec, QC, Canada.; ^5^ Canada Research Chair in Shared Decision Making and Knowledge Translation, Quebec, QC, Canada.

**Keywords:** Patient Engagement, Systematic Reviews, Evaluation Tools, Technology, Artificial Intelligence

## Abstract

Considering the growing recognition of the importance of patient engagement in healthcare decisions, research and delivery systems, it is important to ensure high quality and efficient patient engagement evaluation tools. In this commentary, we will first highlight the definition and importance of patient engagement. Then we discuss the psychometric properties of the patient engagement evaluation tools identified in a recent review on patient engagement in healthcare organization- and system-level decision-making. Lastly, we suggest future directions for patient engagement and its evaluation tools.

## Introduction

Patient engagement in healthcare decisions, healthcare services organizations, health policy development, and in health research has been highlighted in many studies^1-3^ including by patients themselves.^4^ Patient engagement can lead to better health outcomes,^5^ improves quality of care and patient safety,^6^ and helps control health care costs.^7^ The spectrum of patient engagement varies from simply giving them the information (or documentation) to partnership with them by involving them as equal members of the treatment team or of quality improvement activities.^1,2^

Patients’ roles can range from relatively passive to active. In research, for example, active patients and the public engagement refers to all stages of the research process including “prioritization of studies; design and management of studies; data collection and analysis; and dissemination of findings.”^8^ Active partnership among patients, families, their representatives, citizens, health professionals, researchers and decision-makers can improve the responsiveness of the healthcare system to the needs of its users.^1^

However, in spite of efforts to expand patient engagement, formal evaluations of their engagement are rare.^9^ Various frameworks are available for evaluating patient engagement at different levels—local, national, and international.^10,11^ For instance, in Canada, the Canadian Institutes for Health Research’s Supporting Patient-Oriented Research (SPOR) program designed a patient engagement framework in 2014 in collaboration with patient representatives and patient engagement experts at the SPOR patient engagement consultation workshop. This framework was designed to establish key concepts, principles and areas for patient engagement.^10^ However, there is still a knowledge gap about tools for evaluating patient engagement, and about the quality of those tools that have been identified.

## Evaluation Tools

There are a number of evaluation frameworks for patients, public, consumers, and community engagement at different levels. A recent systematic review by Dukhanian and colleagues^12^ followed a rigorous search strategy to review metrics and evaluation tools for patient engagement in decision making. Their review included the gray literature, with searches restricted to clinical healthcare delivery organizations and systems. They inductively developed a comprehensive taxonomy of 116 possible evaluation metrics and mapped 23 identified tools for measuring engagement of patients, the public, consumers, and communities into either outcome or process metrics. We used this review as a launchpad to further evaluate the psychometric properties of the identified tools, and as the authors themselves pointed out, the psychometric reporting was poor.

The review found 21 studies reporting on 23 tools. We used the COSMIN checklist^13^ to evaluate the process of development and/or validation of these tools. Six out of the 21 studies had the objective of developing a tool^14-19^ and one had the objective of validating a tool (responsiveness parameter).^20^ These studies identified relatively few measurement properties. Good measurement properties for tools should include structural validity, internal consistency, reliability, measurement error, criterion validity, hypotheses testing for construct validity, measurement validity, and responsiveness.^21-23^ The original studies on the development and/or validation of patient engagement tools did not report enough information on measurement properties. Even the one study for which validating a tool was a stated objective reported only one measurement property,^20^ and only one out of the six studies whose stated objective was to develop a tool reported a measurement property.^16^ Among the other studies (ie, those without stated validation or development objectives), four reported at least one measurement property.^24-27^

## Discussion and Future Research in Patient Engagement

There is growing attention to patient engagement in the design and implementation of healthcare services and in decision making. However, still more effort is required to both engage patients and to evaluate this engagement. Here we summarize three suggestions for future studies in patient engagement.

*First,* both the systematic review by Dukhanian and colleagues and our evaluation of the psychometric properties of the tools it identified confirm the lack of studies on tool development and/or on validation process that use rigorous methods as well as a lack of detailed reporting of each step. A systematic review by Boivin and colleagues also suggested the scientific rigour of such tools in research and health system decision making should be improved.^28^ For future studies, we suggest that the tool development and validation process, at any level, follow rigorous methods and report each step in detail. The aim of the tool should be clearly stated in future studies. As suggested in a methodological article on tools in health or social sciences,^29^ three aims for patient engagement evaluation tools can be distinguished: (*i*) to discriminate between levels of patient engagement; (*ii*) to predict the results of research that engages patients; and (*iii*) to evaluate the change in patient engagement over time. To be rigorous, some psychometric properties (eg, internal consistency, temporal stability) should be considered for all tools regardless of their aims, while others are specific to the defined aim.^30^ For example, responsiveness is a validation parameter important for tools aiming to evaluate a change over time.^30,31^

*Second,* the taxonomy of possible engagement evaluation metrics developed by Dukhanian and colleagues could be validated with international experts, including with patient-evaluators and public representatives, using group-based approaches such as a Delphi-type exercise^32^ to produce a consensually agreed structured taxonomy. This method permits stakeholders to give their opinions about the formulation and relevance of different concepts as well as their definitions.

*Third,* as mentioned earlier, the purpose of evaluation tools can be to evaluate the change in patient engagement over time. Current fast-growing technologies such as artificial intelligence (AI) may facilitate achieving this goal in the future. AI could be used to evaluate patient engagement over time, based on the systematically collected data, or even foster patient engagement using patient records or databases on successful patient engagement interventions to predict facilitating factors for active patient engagement in a targeted population.

It is important that patients be involved in the design and implementation of such technologies making sure their preferences and needs are considered.^33^ This will raise the need for development and validation of evaluation tools for patient engagement in AI-related research in health. More investigation is required into how to use AI efficiently and meaningfully in engaging patients and into evaluating their engagement at different levels.

## Acknowledgements

FL holds Canada Research Chair in Shared Decision Making and Knowledge Translation. Authors also thank Health and Social Services Systems, Knowledge Translation and Implementation Component of the Quebec SPOR-SUPPORT Unit.

## Ethical issues

Not applicable.

## Competing interests

Authors declare that they have no competing interests.

## Authors’ contributions

SAR and HTVZ conceived and designed the study. SAR and HTVZ conducted the evaluations and analysis. All authors contributed to the interpretation. SAR was major contributor in the writing of the manuscript. All authors revised the manuscript, and read and approved the final version.

## Authors’ affiliations

^1^Department of Family Medicine, Faculty of Medicine, McGill University, Montreal, QC, Canada. ^2^Health and Social Services Systems, Knowledge Translation and Implementation Component of the Quebec SPOR-SUPPORT Unit, Université Laval, Quebec, QC, Canada. ^3^Université Laval Primary Care Research Centre (CERSSPL-UL), Université Laval, Quebec, QC, Canada.^4^Department of Family Medicine and Emergency Medicine, Faculty of Medicine, Université Laval, Quebec, QC, Canada. ^5^Canada Research Chair in Shared Decision Making and Knowledge Translation, Quebec, QC, Canada.
